# Pattern of Maternal Complications and Low Birth Weight: Associated Risk Factors among Highly Endogamous Women

**DOI:** 10.5402/2012/540495

**Published:** 2012-09-08

**Authors:** Abdulbari Bener, Khalil M. K. Salameh, Mohammad T. Yousafzai, Najah M. Saleh

**Affiliations:** ^1^Department of Epidemiology and Medical Statistices, Hamad General Hospital, Hamad Medical Corporation, Qatar; ^2^Department Evidence for Population Health Unit, School of Epidemiology and Health Sciences, The University of Manchester, Manchester, UK; ^3^Departments of Public Health and Medical Education, Weill Cornell Medical College, P.O. Box 3050, Doha, Qatar; ^4^NICU Unit, Women's Hospital, Hamad Medical Corporation, Qatar; ^5^Obstetrics and Gynecology Department, Women's Hospital, Hamad Medical Corporation, Qatar

## Abstract

*Objective*. The objective of the study was to examine the pattern of low birth weight LBW, maternal complications, and its related factors among Arab women in Qatar. *Design*. This is a prospective hospital-based study. *Setting*. The study was carried out in Women's Hospital, Doha. *Subjects and Methods*. Pregnant women in their third trimester were identified in the log book of Women's Hospital and recruited into the study during first week of January 2010 to July 2011. Only 1674 (out of 2238) Arab women (74.7%) consented to participate in this study. Data on clinical and biochemistry parameters were retrieved from medical records. Follow-up data on neonatal outcome was obtained from labor room register. *Results*. The incidence of LBW (<2500 g) was 6.7% among Arab women during 2010 in Qatar. Distribution of gestational diabetes mellitus (GDM), antepartum hemorrhage (APH), maternal anemia, premature rupture of membrane (PROM), maternal occupation, parity, sheesha smoking, and parental consanguinity were significantly different (*P* < 0.05) between mothers of LBW and normal birth weight NBW (≥2500 g) babies. Multivariable logistic regression analysis revealed that previous LBW, consanguinity, parity, smoking shesha, GDM, APH, anemia, PROM, maternal occupation, and housing condition were significantly associated with LBW adjusting for gestational age. *Conclusion*. Maternal complications such as GDM, APH, anemia, PROM, and smoking shesha during pregnancy are significantly increasing the risk of LBW outcome. Screening and prompt treatment for maternal complications and health education for smoking cessation during routine antenatal visits will help in substantial reduction of LBW outcome.

## 1. Introduction

Complications of pregnancy and child birth are the leading causes of disability and death among women of reproductive age in developing countries accounting for at least 18% of the global burden of disease in this age group [1]. Similarly, the pattern of leading causes of maternal death and disability is closely linked to poor maternal health during pregnancy, inadequate care during delivery, and lack of new born care [1]. Every year, almost 8 million still births and early neonatal deaths occur. In addition to maternal deaths, more than 50 million women experience maternal health problems annually [2]. In underdeveloped countries, those LBW and maternal complications often pose an immediate financial burden on women and their households.

With the advent of modern medicine, labour and delivery have become much safer for both mother and baby, but complications still occur. The third trimester, which is 28th week onwards till delivery, is like the last lap of the pregnancy journey. This period is a period where certain obstetric and medical problems can develop and a crucial phase for baby's weight gain. The adverse events that occur during pregnancy influence the health of the infant that may result in the neonatal outcome. The main potential complications that affect the mother during the third trimester are pregnancy-induced hypertension (preeclampsia and eclampsia), gestational diabetes, anaemia, bleeding, placenta praevia, abruptio placenta, vasa praevia, preterm labour, premature rupture of membrane, and so forth.

The health of a pregnant woman has a profound effect on the health of the developing fetus and new born [3]. According to World Health Organization, congenital malformations are now the 3rd leading cause of infant mortality, accounting for 12.7% of early neonatal mortality [4]. It was reported that 70–80% of all neonatal mortality and morbidity is due to preterm birth which is one of the major clinical problems in obstetrics and neonatology [5]. Infants born to women with diabetes are at increased risk for adverse birth outcomes [6, 7]. More recently reported study on gestational diabetes is considered to be a major public health problem associated with higher perinatal mortality and morbidity rates [6, 7].

The objective of the study was to examine the pattern of low birth weight, maternal complications, and related factors that occur in the third trimester of Arab women.

## 2. Subjects and Methods

**Figure 1:**
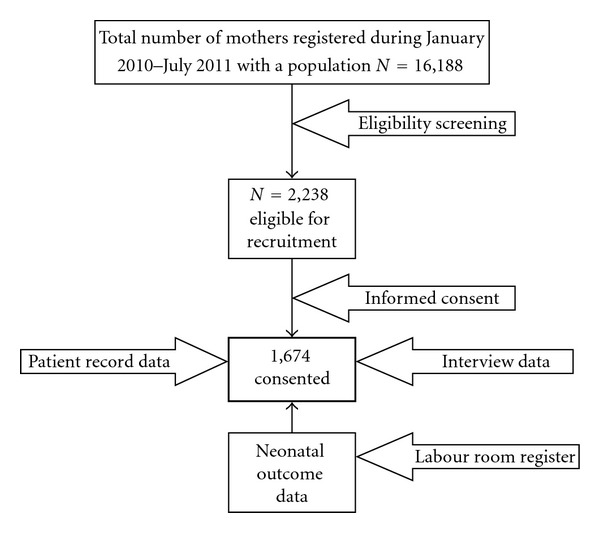
Flow diagram of representative sample studied *N* = 1,674.

This is a prospective hospital-based study conducted among the Arab pregnant women in third trimester during January 2010 to July 2011. The study was based on the log book of the Women's Hospital which registers all the pregnant women visiting antenatal clinics of Women's Hospital, Hamad Medical Corporation. The research assistants screened the log book of Women's Hospital during the study period and prepared a list of 2,238 Arab pregnant women above 24 weeks of gestation with any maternal complication. All the eligible women on the prepared list (*N* = 2,238) were approached and consent was sought. Only 1674 women (74.7%) consented to participate in the study (refer to Figure 1). In 2010, there were a total of 16,188 deliveries in the Women's Hospital. Our study sample included 1674 pregnant women which is 10.3% of the mothers delivered. The study was approved by the Hamad Medical Corporation prior to commencing data collection. Each participant was provided with brief information about the study and was assured of strict confidentiality.

Data was collected through face-to-face interview by qualified nurses using a validated questionnaire in the local language. The questionnaire covered sociodemographic characteristics of the pregnant women, family and medical history. The questionnaire was pilot-tested on 100 randomly selected pregnant women for the validity. The investigators made necessary corrections and modifications after considering the minor differences and discrepancies that had been found during the pilot study. Data on maternal complications of each patient were retrieved from the medical record file. In addition, follow-up data on pregnancy and neonatal outcome of each woman were obtained from the labour room register immediately after delivery (Figure 1).

The study was approved by the IRB of Research Ethics Committee of Hamad Medical Corporation (HMC-MRC) and by the IRB of Weill Cornell Medical College (WCMC-Q).

Data were analyzed using SPSS version 19. Student's *t*-test was used to ascertain the significance of differences between mean values of two continuous variables. Fisher Exact and Chi-Square tests were performed to test for differences in proportions of categorical variables between two or more groups. Univariate logistic regression analyses were carried out to identify possible predictors of LBW (<2500 g). Adjusted odds ratios for all variables that were significantly associated with LBW were computed using a multiple logistic regression model for controlling the simultaneous confounding effects of possible confounders. Hosmer-Lemeshow goodness-of-fit test was used to assess the model adequacy. All statistical tests were two-sided, and *P* < 0.05 was considered statistically significant.

## 3. Results

The incidence of low birth weight (<2500 g) was 6.7% during the period 2010 in Qatar.  Table 1 shows distribution of sociodemographic characteristics across LBW and NBW babies. There was no statistical difference between LBW and NBW in terms of nationality, maternal age, maternal education, household monthly income, place of living, and father's occupation. However, mother's occupation (house wife or working) and type of residence (villa, apartment, or population house) were significantly different (*P*-0.009 and 0.033, resp.) between LBW and NBW babies. Almost three quarters of the LBW babies were born to mothers who were housewives as compare to almost a quarter of LBW babies to mothers working outside.

Table 2 shows distribution of maternal and obstetric characteristics across LBW and NBW babies. There was a significant difference between mothers with LBW and NBW babies in terms of consanguinity (*P* = 0.021), parity (*P*-0.038), previous LBW (*P* = 0.006), and shesha smoking (*P* = 0.024). No significant difference was observed in terms of maternal BMI, previous LBW, previous abortion, and antenatal care.

Table 3 shows distribution of maternal complications across LBW and NBW babies. There was a significant difference in terms of GDM (*P*-0.032), APH (*P*-0.046), anemia (*P*-0.050), PROM (*P*-0.007), and gestational age groups (<0.001).

Table 4 shows determinants of LBW based on multivariable logistic regression analysis. Previous LBW (Adj. OR 1.9; 95% CI 1.3–3.1), consanguinity (Adj. OR 1.6; 95% CI 1.1–2.3), parity (2-3 versus <2, Adj. OR 1.8; 95% CI 1.1–3.1 and ≥4 versus <2, Adj. OR 2.0; 95% CI 1.2–3.3), smoking shesha (Adj. OR 2.0; 95% CI 1.1–3.7), GDM (Adj. OR 1.8; 95% CI 1.1–3.4), APH (Adj. OR 1.6; 95% CI 1.1–2.5), anemia (Adj. OR 1.6; 95% CI 1.0–2.4), PROM (Adj. OR 2.3; 95% CI 1.2–4.2), maternal occupation (working versus house wife, adjusted OR 0.6; 95% CI 0.4–0.9), and housing condition (apartment versus villa, Adj. OR 0.6; 95% CI 0.2–1.4 and popular house versus villa, Adj. OR 1.5; 95% CI 1.1–2.3) were significantly associated with LBW adjusting for gestational age.

## 4. Discussion

The present study highlighted the potential maternal complications that affected the mother during the third trimester and its effect on neonatal birth weight among Arab women in Qatar. It is important to identify the risk factors early in the prenatal period so that appropriate interventions are established to ensure the well-being of the mother and child. In the study sample, majority of the pregnant women with low monthly household income experienced more complications during pregnancy (38.2%) with a higher frequency among expatriate Arab women (41.7%) than Qatari women (33.8%). Most of them were housewives (60.8%) and with university degree (42%). Also, advancing maternal age especially aged 35 years or older has been accepted to have more risks from both the maternal and fetal perspectives.

The study findings revealed that the obstetrical complications as well as neonatal morbidity were higher among older women above 35 years. Previous studies suggested that women aged over 35 years are at increased risk for obstetrical complications as well as perinatal morbidity and mortality [8, 9]. The leading maternal complications were significantly higher among women aged 35 years and above compared to younger women below 35 years; mainly gestational diabetes (20.8% versus 13.4%; *P* < 0.001), gestational hypertension (21.6% versus 15.2%; *P* = 0.003), and antepartum haemorrhage (17.9% versus 13.7%; *P* = 0.042). The modern infertility treatment has increased the number of women able to become pregnant at advanced age. The outcome of these pregnancies raised significant concern because older age is associated inherently with higher incidence of chronic diseases. Another study by Üstün et al. [10] found similar results that complications of pregnancy were higher in the older group.

Harrison [11] stated in their study that one quarter of all adult women living in the developed world currently suffer from a short- or long-term illness related to pregnancy and childbirth. Roberts and Cooper [12] reported that gestational hypertension and severe preeclampsia are leading causes of maternal and fetal/neonatal morbidity and mortality which is similar to our study results. Maternal anemia is considered as a risk factor for adverse pregnancy outcome [13]. Estimates from the World Health Organization report [14] that 35% to 75% of pregnant women in developing countries and 18% of women from industrialized countries are anemic. Low birth weight in anemic women has been observed in the present study (24.1%). There is substantial amount of evidence showing that maternal anemia in pregnancy can result in low birth weight subsequent to preterm delivery [15].

It was documented that the prevalence of gestational diabetes is increasing all over the world [6, 16–18] and the risks include an increased risk of macrosomia, birth injuries, and caesarean deliveries [6, 16, 18]. Our rate of macrosomia (42.3%) is strikingly similar to a prospective study in The Netherlands [19]. In Saudi Arabia [20], it was found that congenital malformations and neonatal jaundice were the most common problems among babies of gestational diabetic mothers.

The current study has shown that maternal complications are major contributors to neonatal complications. A high-risk pregnancy with maternal complications was observed among women of advanced age. Women with severe maternal complications like gestational hypertension, maternal anaemia, gestational diabetes, antepartum haemorrhage had a greater chance to have a poor neonatal outcome. Gestational diabetes increased the risk of macrosomia and caesarean delivery. The high consanguinity rate, advanced maternal age, and past obstetric risk of congenital anomalies in studied women might have increased the risk of congenital anomalies in newborns of older women. Low birth weight was found higher in maternal anaemia and antepartum haemorrhage.

In last, the study highlighted the association of maternal complications, lack of family planning practices (parity > 2), and maternal shesha smoking with delivering low birth weight babies. Early identification and prompt treatment for maternal complications and health education for smoking cessation during routine antenatal visits will help in substantial reduction of LBW outcome. Mass awareness should be created by utilizing mass and print media regarding early signs of maternal complications, smoking cessation, and essential family planning practices to promote primary prevention of LBW in Qatar.

## Figures and Tables

**Table 1:** Sociodemographic characteristics among mothers of LBW and NBW babies in Qatar (*N* = 1674).

Variable	LBW *n* = 112 (%)	NBW *n* = 1562 (%)	*P* value^∗^
Nationality			
Qatari	56 (50.0)	728 (46.6)	0.487
Non-Qatari	56 (50.0)	834 (53.4)	
Maternal age at delivery			
<25 yrs	20 (17.9)	246 (15.7)	0.743
25–34 yrs	56 (50.0)	766 (49.0)	
≥35 yrs	36 (32.1)	550 (35.2)	
Maternal education			
Primary or below	28 (25.0)	267 (17.1)	0.120
Intermediate	10 (8.9)	139 (8.9)	
Secondary and above	74 (66.1)	1156 (74.0)	
Mother's occupation			
House wife	81 (72.3)	935 (59.9)	**0.009**
Working outside	31 (27.7)	627 (40.1)	
Household income (*∫*)			
<10,000	56 (50.0)	731 (46.8)	0.512
≥10,000	56 (50.0)	831 (53.2)	
Type of residence			
Villa	67 (59.8)	1027 (65.7)	**0.033**
Apartment	5 (4.5)	135 (8.6)	
Popular house	40 (35.7)	1027 (65.7)	
Place of living			
Urban	94 (83.9)	1334 (85.4)	0.670
Semi urban	18 (16.1)	228 (14.6)	
Father's occupation			
Business	21 (18.8)	263 (16.8)	0.263
Retired	20 (17.9)	230 (14.7)	
Professional	11 (9.8)	260 (16.6)	
Others	60 (53.6)	809 (51.8)	

*∫*: Qatari Riyal, LBW: low birth weight (birth weight less than 2500 g), NBW: normal birth weight (2500 g or more).

^∗^Two-sided *P* values based on Chi-Square test.

**Table 2:** Maternal and obstetric characteristics among mothers of LBW and NBW babies in Qatar (*N* = 1674).

Variable	LBW *n* = 112 (%)	NBW *n* = 1562 (%)	*P* value^∗^
Body mass index (Kg/m^2^)			
Normal	46 (41.1)	779 (49.9)	0.136
Overweight	32 (28.6)	420 (26.9)	
Obese	34 (30.3)	323 (23.2)	
Consanguinity in parents			
Yes	45 (40.2)	536 (34.3)	**0.0** **21**
No	67 (59.8)	1026 (65.7)	
Parity			
<2	19 (17.0)	434 (27.8)	**0.038**
2-3	32 (28.6)	417 (26.7)	
4–6	57 (50.9)	625 (40.0)	
>6	4 (3.6)	86 (5.5)	
Previous LBW			
Yes	21 (18.8)	158 (10.1)	**0.006**
No	91 (81.3)	1404 (89.9)	
Previous abortion			
Yes	20 (17.9)	207 (13.3)	0.134
No	92 (82.1)	1355 (81.7)	
Previous still birth			
Yes	12 (10.7)	132 (8.5)	0.409
No	100 (89.3)	1430 (91.5)	
Antenatal care			
Yes	90 (80.4)	1210 (77.5)	0.478
No	22 (19.6)	352 (22.5)	
Shesha smoking			
Yes	13 (11.6)	96 (6.1)	**0.024**
No	99 (88.4)	1466 (93.9)	

LBW: low birth weight (birth weight less than 2500 g), NBW: normal birth weight (2500 g or more).

^∗^Two-sided *P* values based on Chi-Square test.

**Table 3:** Maternal complications among mothers of LBW and NBW babies in Qatar (*N* = 1674).

Variable	LBW *n* = 112 (%)	NBW *n* = 1562 (%)	*P* value^∗^
Gestational diabetes mellitus			
Yes	13 (11.6)	311 (19.9)	**0.032**
No	99 (88.4)	1251 (80.1)	
Pregnancy hypertension			
Yes	15 (13.4)	289 (18.5)	0.175
No	97 (86.8)	1273 (81.5)	
Antepartum hemorrhage			
Yes	24 (21.4)	226 (14.5)	**0.046**
No	88 (78.6)	1336 (85.5)	
Anemia			
Yes	27 (24.1)	265 (17.0)	**0.050**
No	85 (75.9)	1297 (83.0)	
Premature rupture of membrane			
Yes	13 (11.6)	85 (5.4)	**0.007**
No	99 (88.4)	1477 (94.6)	
Gestational age (weeks)			
28–31	7 (6.3)	9 (0.6)	**<0.001**
31–36	12 (10.7)	31 (2.0)	
≥36	93 (83.0)	1522 (97.4)	
Preterm delivery			
Yes	23 (20.5)	155 (9.9)	**<0.001**
No	89 (79.5)	1407 (90.1)	

LBW: low birth weight (birth weight less than 2500 g), NBW: normal birth weight (2500 g or more).

^∗^Two-sided *P* values based on Chi-Square test.

**Table 4:** Determinants of low birth weight among newborn babies in Qatar (*N* = 1674).

	Adj. OR (95% CI)	*P* value^∗^
Previous LBW		
No	1	0.006
Yes	1.9 (1.3–3.1)	
Consanguinity		
No	1	0.021
Yes	1.6 (1.1–2.3)	
Parity		
<2	1	0.043
2-3	1.8 (1.1–3.1)	
≥4	2.0 (1.2–3.3)	
Smoking shesha		
No	1	0.026
Yes	2.0 (1.1–3.7)	
Gestational diabetes mellitus		
No	1	0.034
Yes	1.8 (1.1–3.4)	
Antepartum hemorrhage		
No	1	0.048
Yes	1.6 (1.1–2.5)	
Anemia		
No	1	0.050
Yes	1.6 (1.0–2.4)	
Premature rupture of membrane		
No	1	0.009
Yes	2.3 (1.2–4.2)	
Gestational age		
28–31	12.7 (4.6–34.9)	<0.001
31–35	6.3 (3.1–12.7)	
≥36	1	
Maternal occupation		
Housewife	1	0.010
Working outside	0.6 (0.4–0.9)	
Housing condition		
Villa	1	0.045
Apartment	0.6 (0.2–1.4)	
Popular house	1.5 (1.1–2.3)	

Outcome = birth weight (1 = low birth weight, 0 = normal birth weight).

^∗^Two-sided *P* values based on −2 log likelihood ratio statistics.
